# Supplementary material for: Relaxin gene delivery mitigates liver metastasis and synergizes with check point therapy
Source: Nat Commun. 2019 Jul 5;10:2993. doi: 10.1038/s41467-019-10893-8 (PMC6611764; doi:10.1038/s41467-019-10893-8)
Supplement: Supplementary file 1 — Supplementary Information [file 41467_2019_10893_MOESM1_ESM.pdf]

Supporting materials for

Relaxin gene delivery mitigates liver metastasis and synergizes with check point therapy

Hu et al.

Relaxin gene delivery mitigates liver metastasis and synergizes with check point therapy

Mengying Hu[1], Ying Wang[1,2], Ligeng Xu[1, 3], Sai An[1], Yu Tang[4], Xuefei Zhou[1], Jingjing Li[2], Rihe Liu[2], Leaf Huang*[1]

[1]Division of Pharmacoengineering and Molecular Pharmaceutics, Eshelman School of Pharmacy, University of North Carolina, Chapel Hill, NC 27599, USA.

[2]Division of Chemical Biology and Medicinal Chemistry, Eshelman School of Pharmacy, University of North Carolina, Chapel Hill, NC 27599, USA.

[3]Jiangsu Key Laboratory for Carbon-Based Functional Materials & Devices, Institute of Functional Nano & Soft Materials (FUNSOM), Soochow University, Suzhou, 215123, China

[4]Division of Pharmacotherapy and Experimental Therapeutics, Eshelman School of Pharmacy, University of North Carolina, Chapel Hill, NC 27599, USA.

*Corresponding author, leafh@email.unc.edu

Supplementary file includes:

Supplementary Figures 1 to 15.

Supplementary Tables 1 to 2.

Supplementary References 1 to 2.

Supplementary Figure 1:

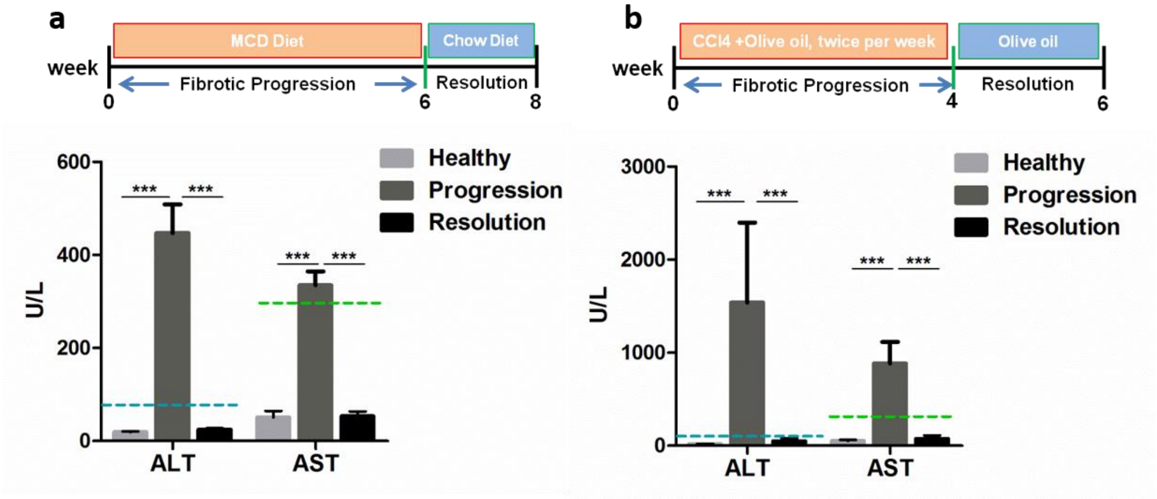

Supplementary Fig.1 Liver injury characterization by ALT and AST at fibrotic progression and resolution in the MCD-induced NASH model (**a**) and CCl4-induced liver fibrosis model (**b**). Blue and green dotted lines represent the upper limit of normal ALT and AST levels, respectively (n=5). Significant differences were assessed using *t* test. Results are presented as mean (S.D.). ***p < 0.001.

Supplementary Figure 2

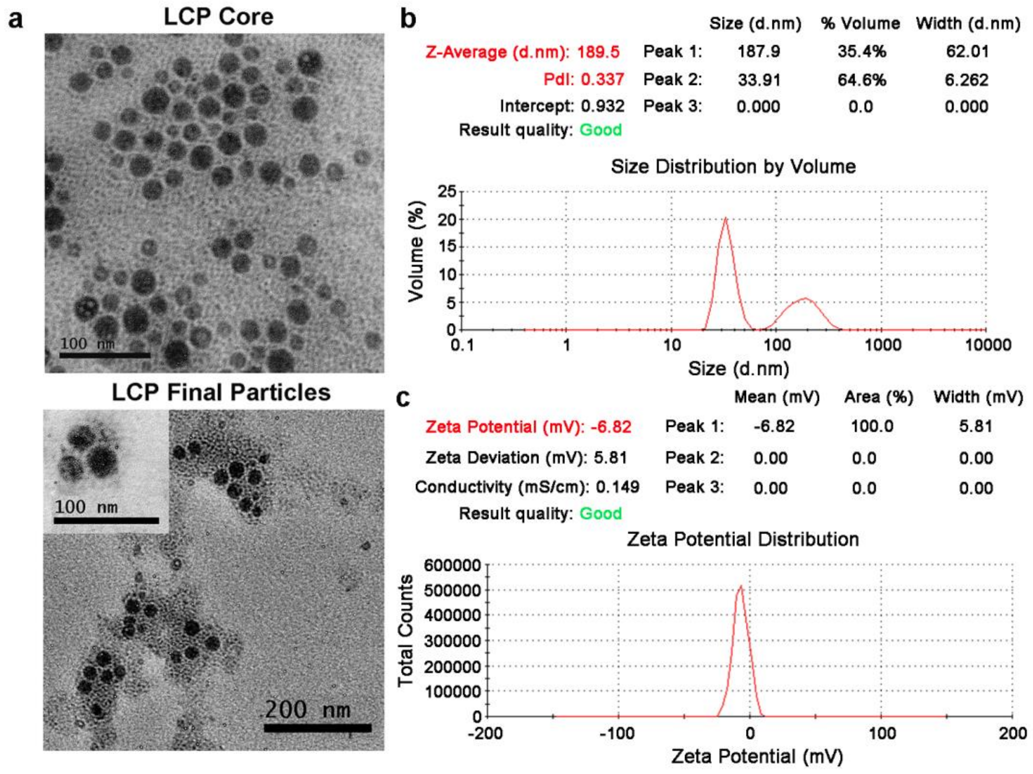

Supplementary Fig. 2 Characterization of the prepared pRLN LCP nanoparticles. (**a)** TEM images. (**b)** Volume average size detected by DLS. (**c)** Zeta potential.

Supplementary Figure 3:

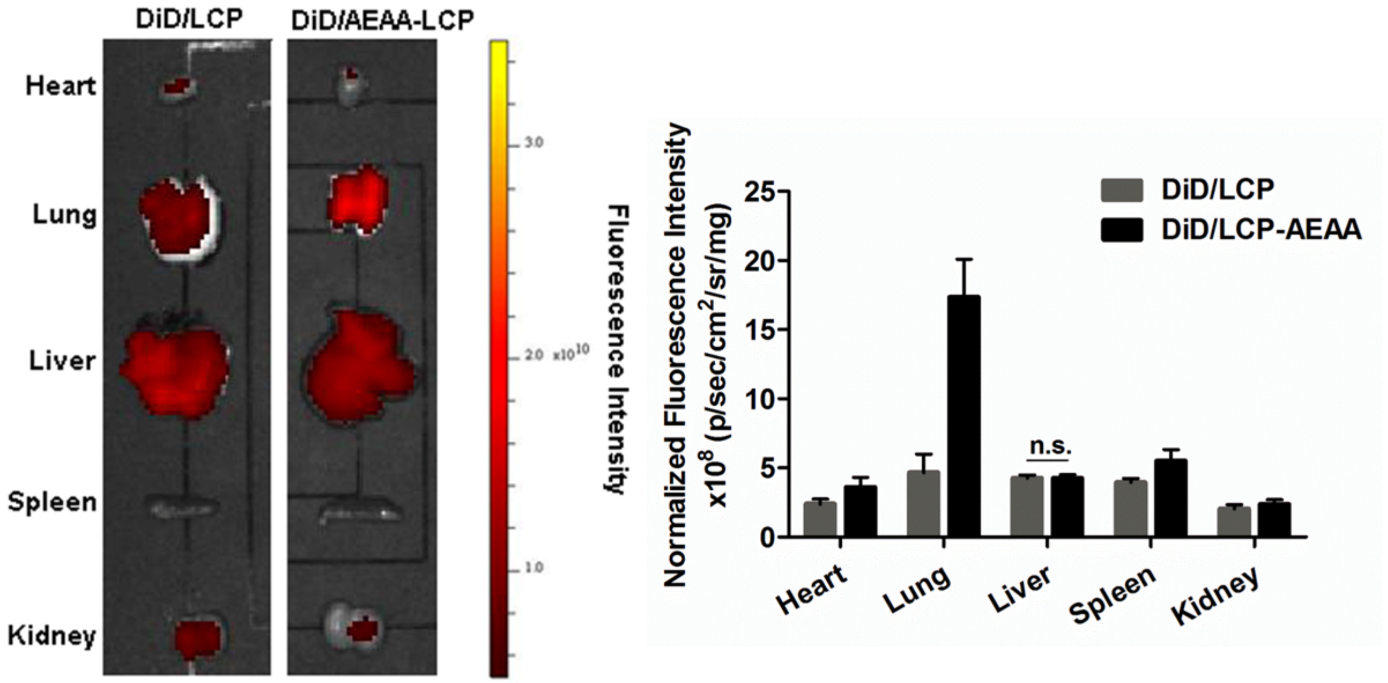

Supplementary Fig. 3 Images and quantitative results of the DiI-loaded LCP (DiD/LCP) or AEAA conjugated LCP (DiD/AEAA-LCP) in major organs at 24 h after injection in healthy BALB/C mice (n = 3). Significant differences were assessed using *t* test. Results are presented as mean (S.D.), n.s., no significance.

Supplementary Figure 4:

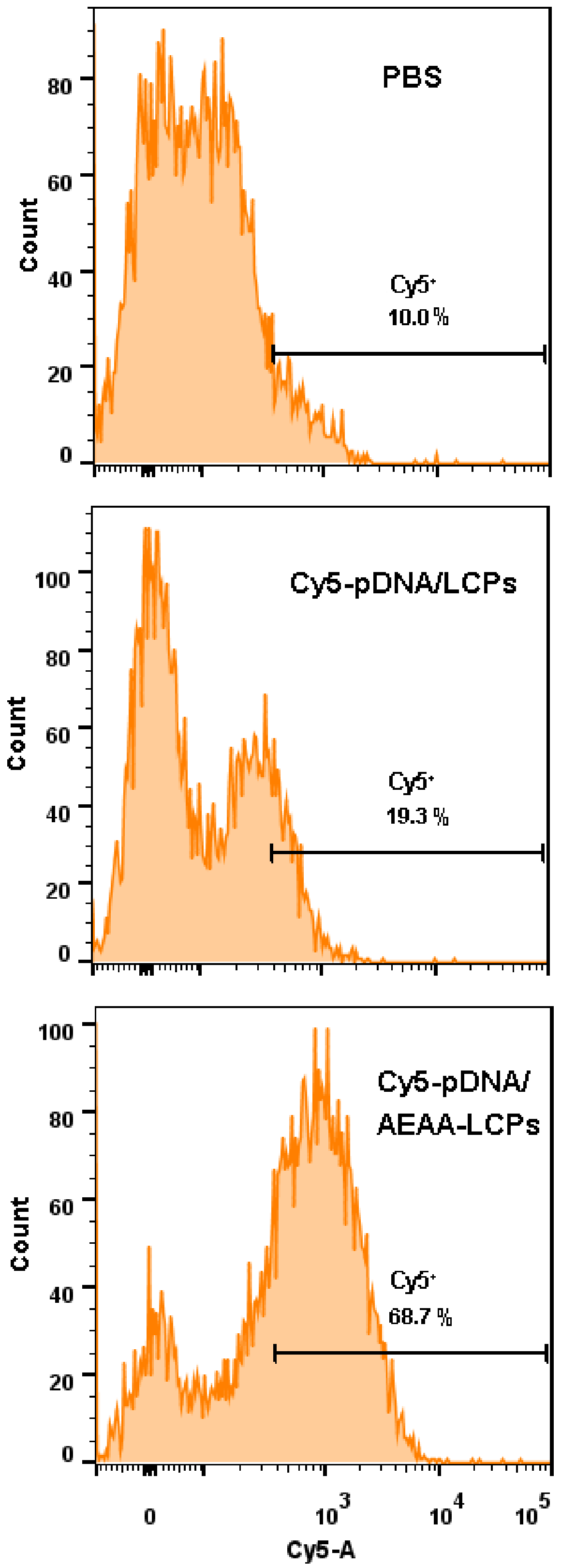

Supplementary Fig. 4 Quantitative uptake of LCPs encapsulating Cy5-labeled pDNA into CT26-FL3 cells with or without AEAA as the targeting ligand by FACS analysis (Thermo Fisher Attune NxT).

Supplementary Figure 5:

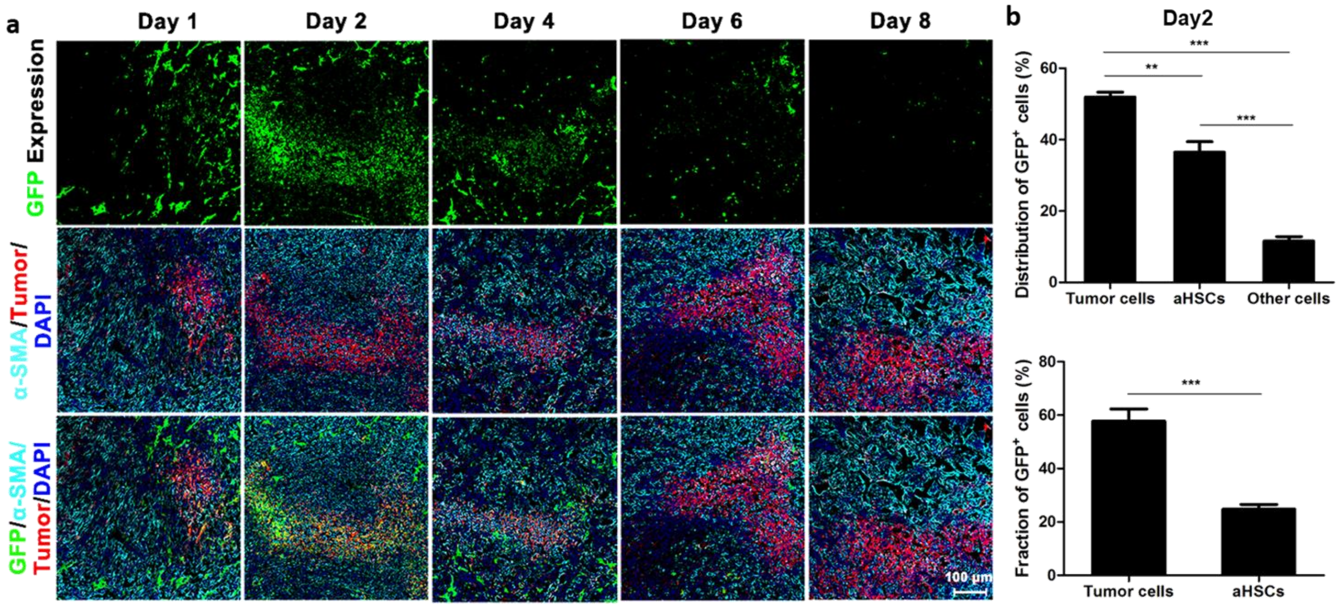

Supplementary Fig. 5 GFP expression in the liver with or without CT26-FL3 metastatic foci. **(a)** GFP expression on day 1, 2, 4, 6, and 8 after 3 injections of pGFP LCP into CT26-FL3 liver metastasis bearing mice. GFP and tumor cells are colored green and red, respectively. Immunofluorescence staining using anti-α-SMA (cyan) and DAPI (blue) were further performed. Bar represents 100 μm. **(b)** Quantification of % tumor cells, % aHSCs, and % other cells in GFP^+ cells (upper penal), as well as $\%GFP^+$ cells in tumor cells and aHSCs (characterized by $\alpha\text{-SMA}^+$) (lower panel) in 5 randomly selected fields per mouse (n=3). Significant differences were assessed using t test. Results are presented as mean (S.D.). $^{**}p < 0.01$, $^{***}p < 0.001$.

Supplementary Figure 6:

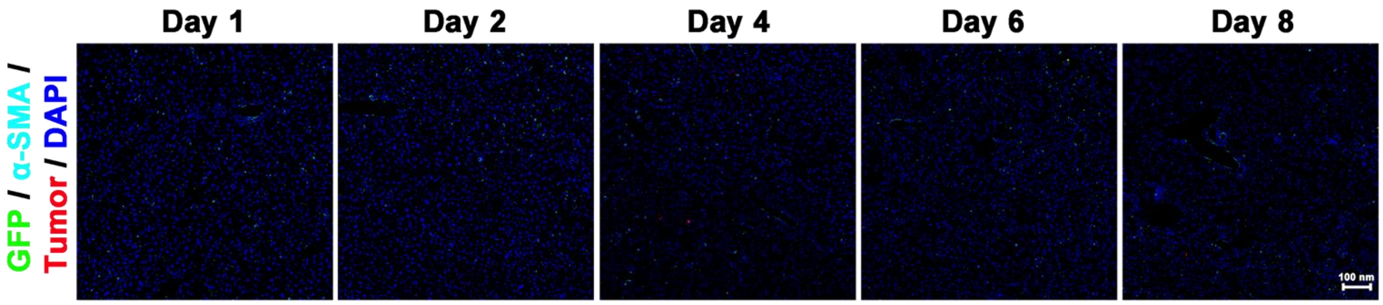

Supplementary Fig. 6 GFP expression in the CT26-FL3 metastasis-free area of the liver after 3 injections of pGFP LCP into CT26-FL3 liver metastasis bearing mice. GFP and tumor cells are colored green and red, respectively. Immunofluorescence staining using anti-α-SMA (cyan) and DAPI (blue) were further performed. Bar represents 100 μm.

Supplementary Figure 7:

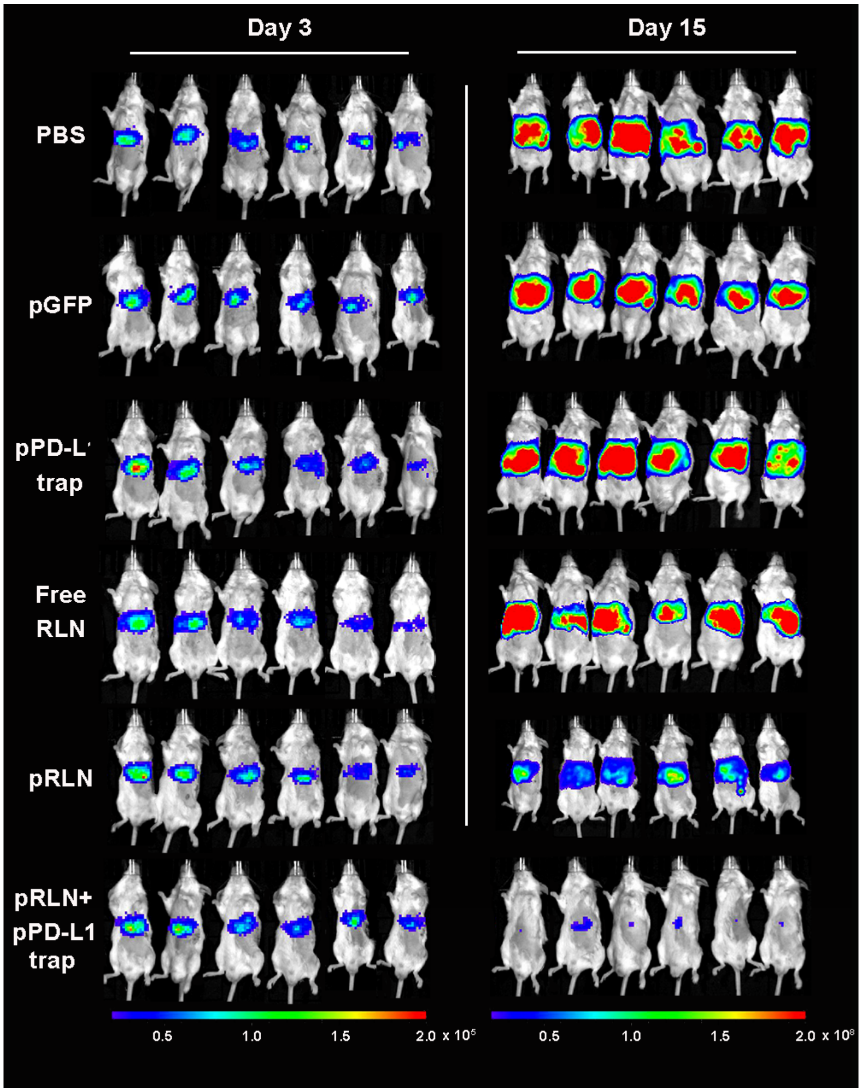

Supplementary Fig. 7 Representative *in vivo* bioluminescence imaging of mice bearing CT26-FL3 liver metastasis receiving various treatments on day 3 and day 15 after tumor inoculation.

Supplementary Figure 8

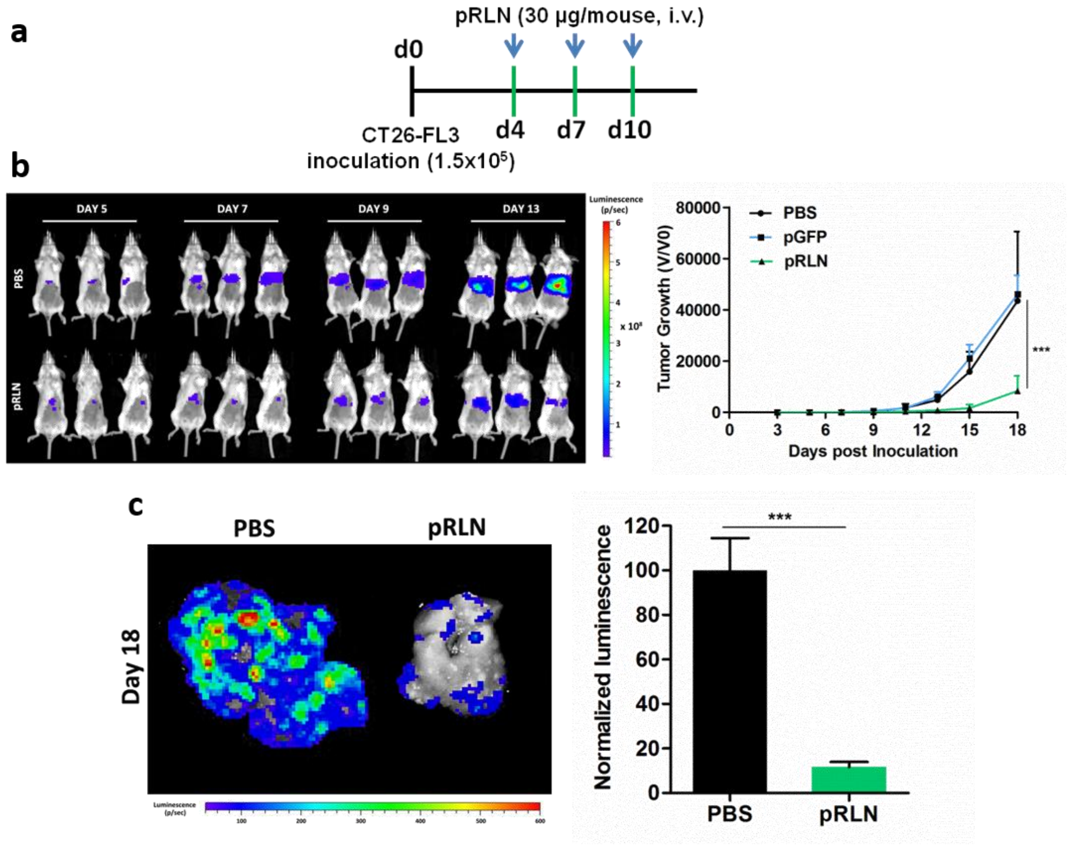

Supplementary Fig. 8 pRLN therapy in female mice bearing CT26-FL3 liver metastasis. **(a)** Tumor inoculation and treatment scheme. **(b)** Representative *in vivo* bioluminescence imaging of mice bearing CT26-FL3 liver metastasis receiving PBS and pRLN LCP treatments and corresponding metastatic growth profile (n=7). **(c)** Imaging and bioluminescence quantification analysis of *ex vivo* CT26-FL3 liver metastasis burden from PBS and pRLN LCP treated groups 18 days after tumor inoculation (n=3). Significant differences were assessed in **b** using two-way ANOVA with multiple comparisons, in **c** using *t* test. Results are presented as mean (S.D.), *p < 0.05, **p < 0.01, ***p < 0.001.

Supplementary Figure 9

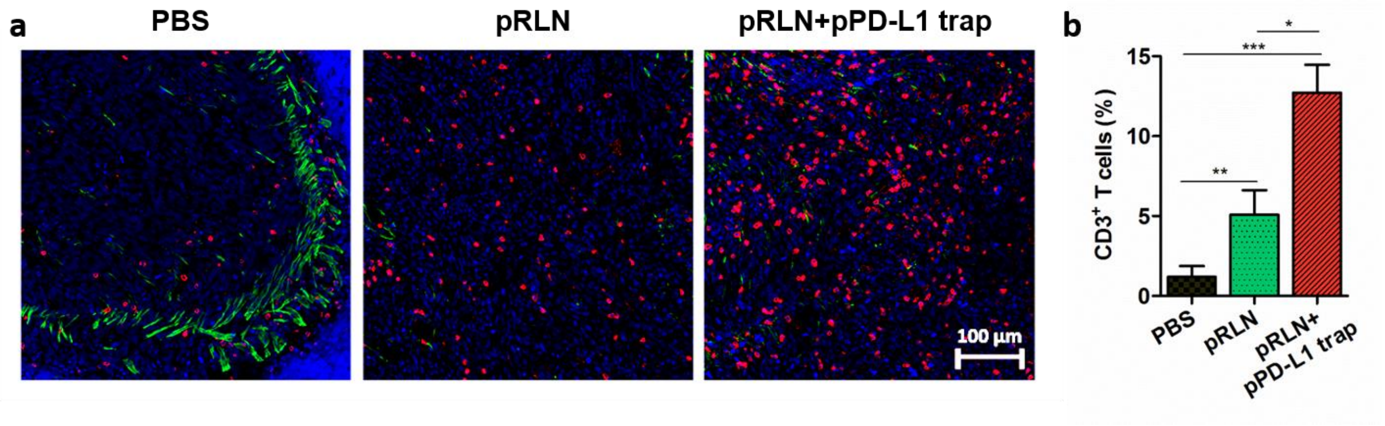

Supplementary Fig. 9 CD3$^+$ T cell penetration into CRC liver metastasis. **(a)** Immunofluorescence staining of CT26-FL3 metastatic livers from PBS, pRLN LCP, and pRLN+pPD-L1 trap LCP treatment groups on day 13 using anti-CD3 (red), α-SMA (green), and DAPI (blue). **(b)** Positive ratios were quantified in 5 randomly selected fields per mouse (n=3). Bar represents 100 μm. Significant differences were assessed using *t test*. Results are presented as mean (S.D.). *$p < 0.05$, **$p < 0.01$, ***$p < 0.001$.

Supplementary Figure 10

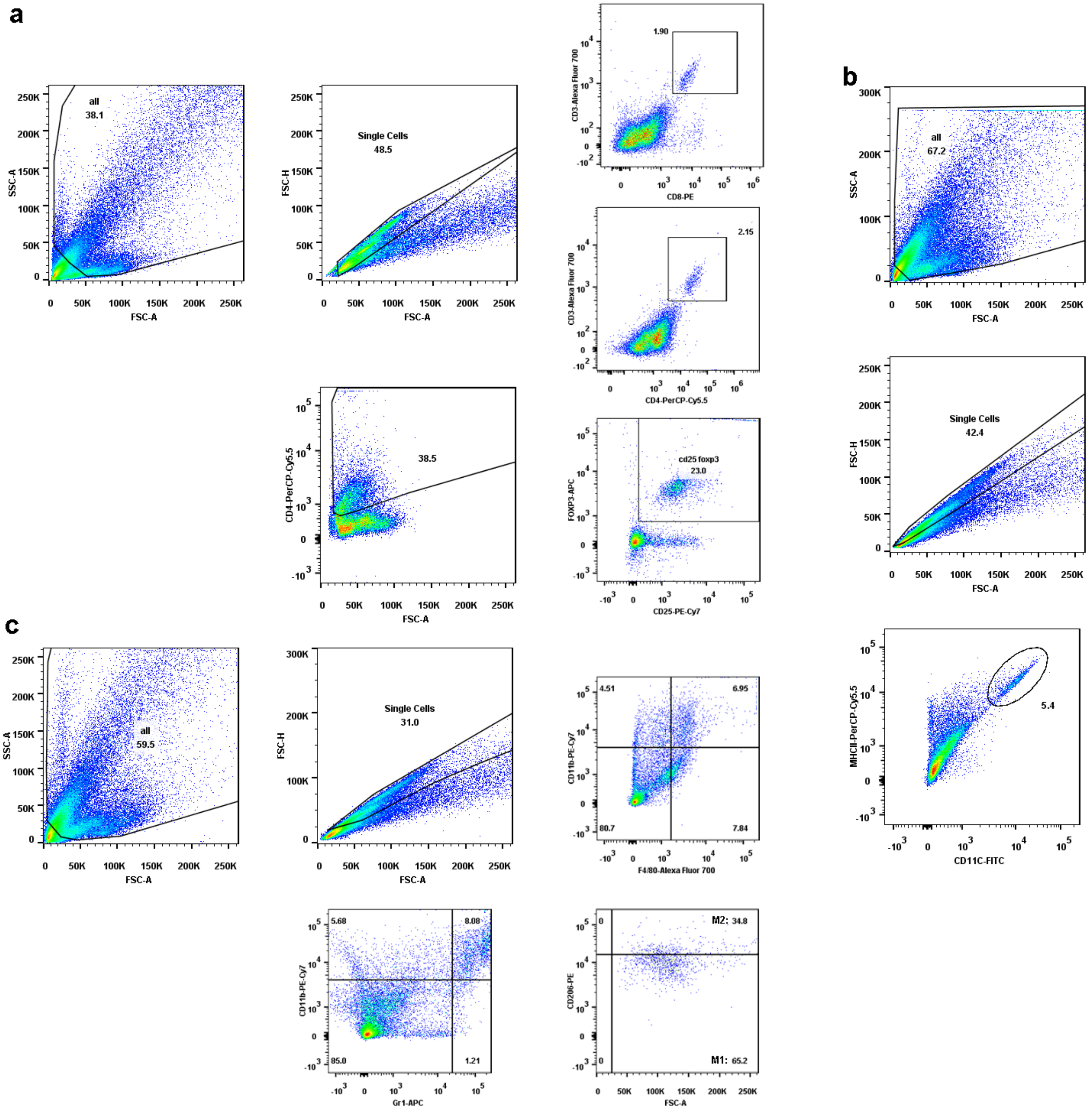

Supplementary Fig. 10 Gating strategies used for flow cytometry analysis of immune cell populations in the metastatic liver. (a) Gating strategy to analyze CD4 ($CD3^+CD4^+$), CD8 ($CD3^+CD8^+$), and Treg ($CD4^+CD25^+FOXP3^+$) cells in the CT26-FL3 metastatic liver after different treatments presented on Fig. 2b, $CD4^+$, $CD8^+$, $CD4^+/CD8^+$ ratio, and Treg cells subpanels. (b) Gating strategy to analyze activated DC ($CD11C^+MHCII^+$) cells in the CT26-FL3 metastatic liver after different treatments presented on Fig. 2b, activated DCs subpanel. (c) Gating strategy to analyze macrophages ($CD11b^+F4/80^+$), M2 macrophages ($CD11b^+F4/80^+CD206^+$), and MDSCs ($CD11b^+Cr\text{-}1^+$) in the CT26-FL3 metastatic liver after different treatments presented on Fig. 2b, M1/M2 ratio and MDSCs subpanels.

Supplementary Figure 11

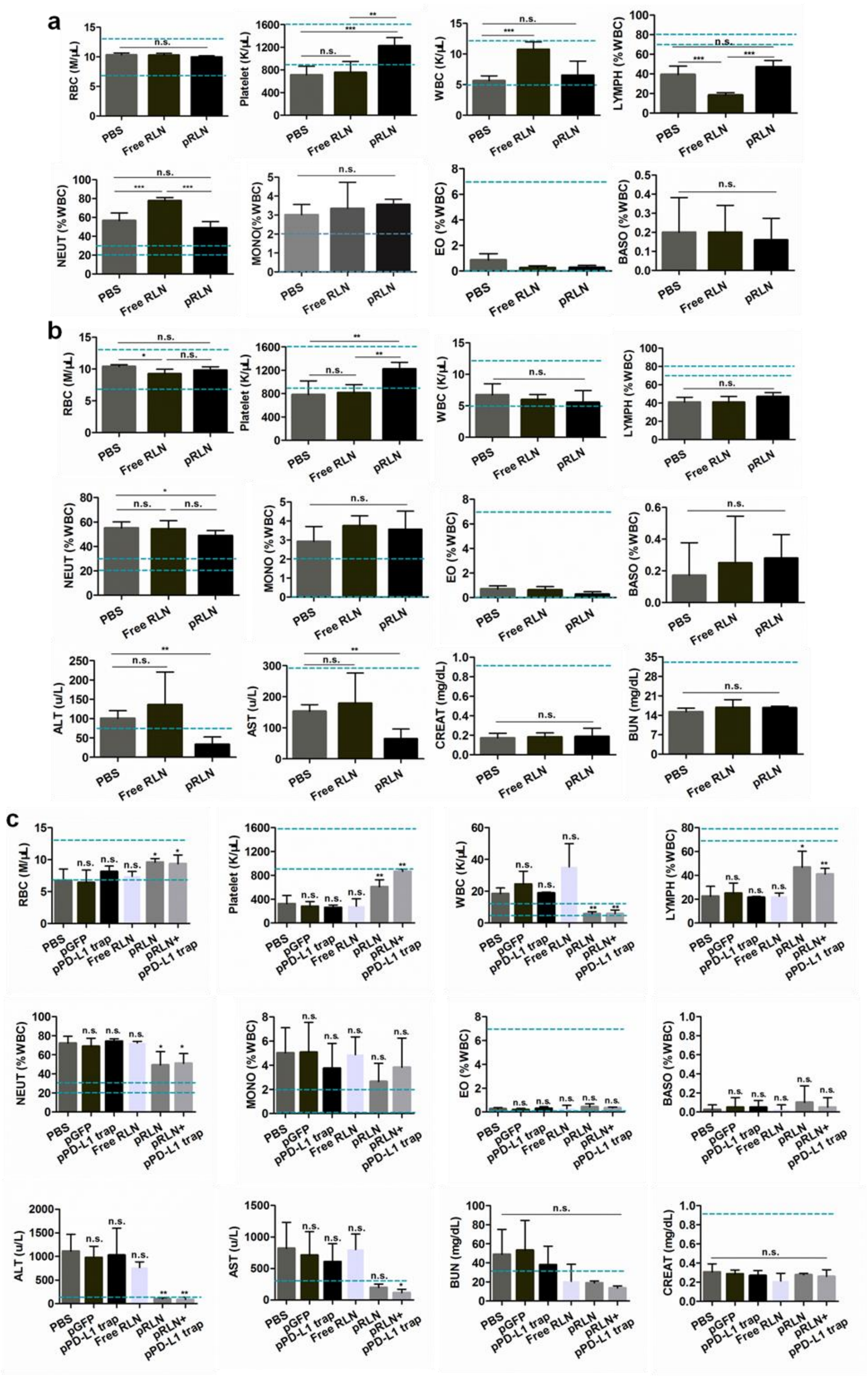

Supplementary Fig. 11 Complete blood count and blood chemistry analysis of CT26-FL3 liver metastasis bearing mice after various treatments 2h (**a**), 24 h (**b**) or 8 days (**c**) after the last injection (n = 4). Blue dotted lines represent the normal range of each indicator. Significant differences were assessed using *t test*. Results are presented as mean (S.D.). *p < 0.05, **p < 0.01, ***p < 0.001, n.s., no significance. The p values of individual groups in (**c**) were calculated by comparing to the PBS control.

Supplementary Figure 12

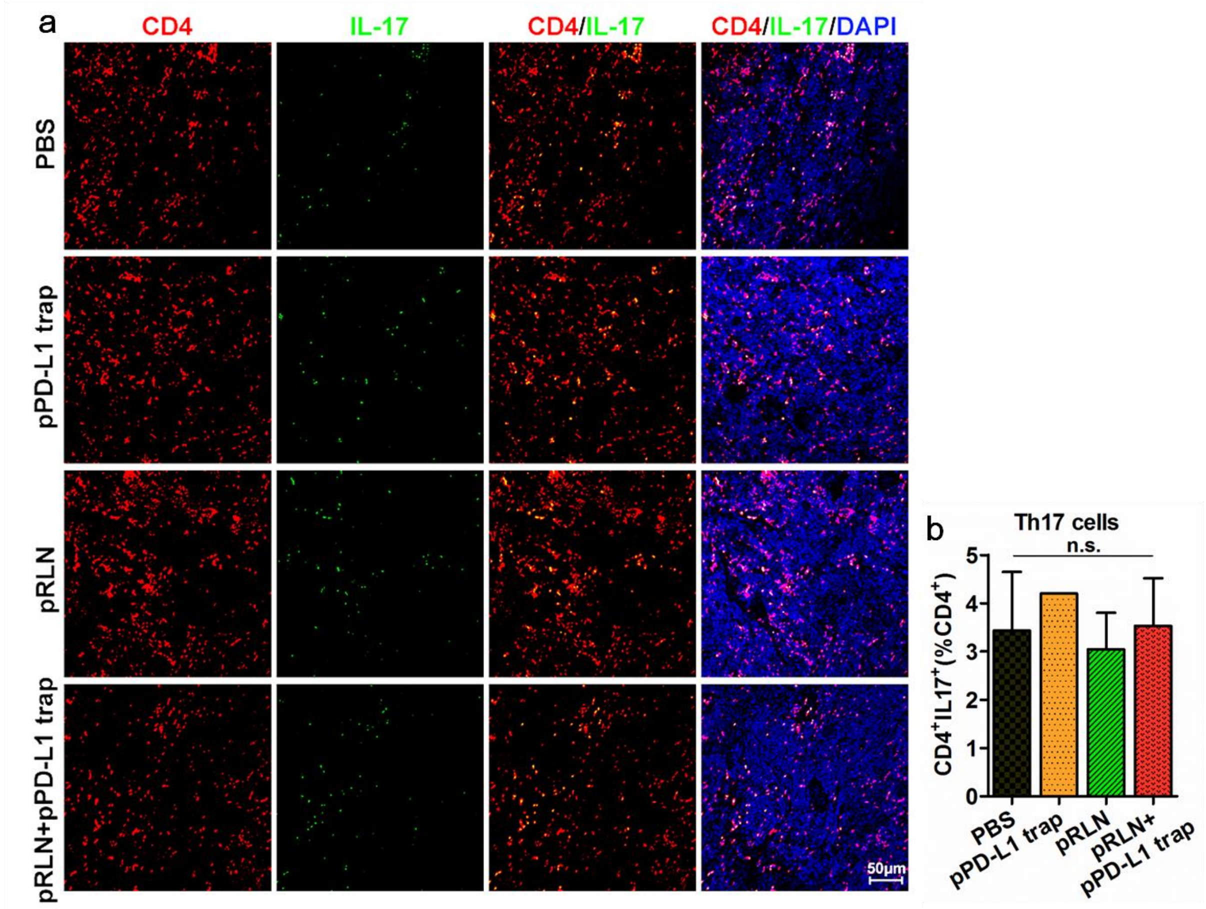

Supplementary Fig. 12 Th17 cells in the spleen of CT26-FL3 liver metastasis bearing mice. **(a)** Immunofluorescence staining of Th17 cells ($CD4^{+}IL\text{-}17^{+}$ T cells) in the spleen from PBS, pPD-L1 trap, pRLN, and pRLN+pPD-L1 trap treatment groups on day 13 using anti-CD4 (red), anti-IL-17 (green), and DAPI (blue). **(b)** %Th17 cells in $CD4^{+}$ cells were quantified in 5 randomly selected fields per mouse (n=3). Bar represents 50 μm. Significant differences were assessed using t test. Results are presented as mean (S.D.). n.s., no significance.

Supplementary Figure 13

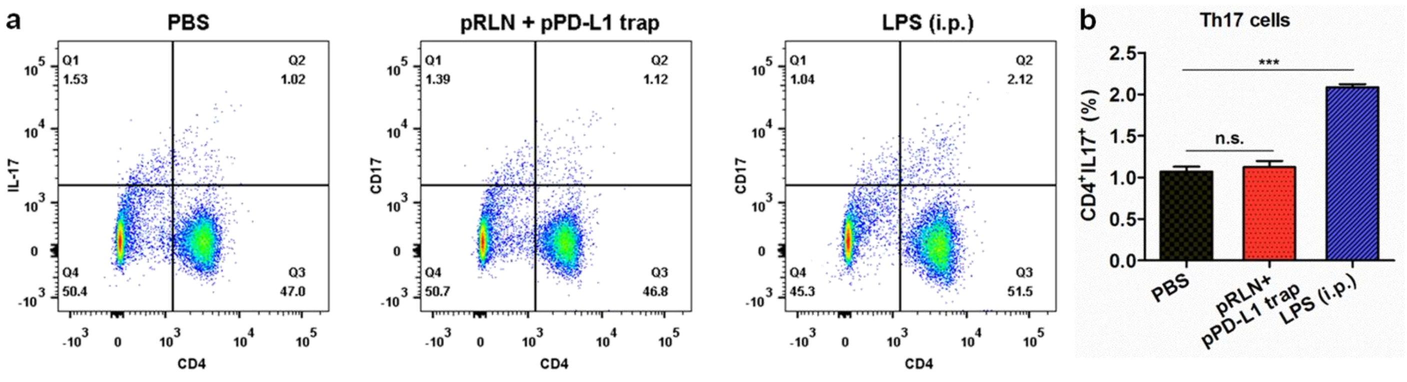

Supplementary Fig. 13 Flow cytometry analysis of Th17 cells in the spleen of C57BL/6 male mice bearing KPC liver metastasis receiving PBS or pRLN+pPD-L1 trap treatments on day 14. Intra-splenic Th17 cells in the spleen of healthy mice receiving a single dose of LPS (50μg, i.p.) were used as a positive control. **(a)** Representative scatter plots and (b) Quantitative analysis of $CD4^{+}IL\text{-}17^{+}$ cells in the spleen (n=4). Significant differences were assessed using t test. Results are presented as mean (S.D.). ***$p < 0.001$, n.s., no significance.

Supplementary Figure 14

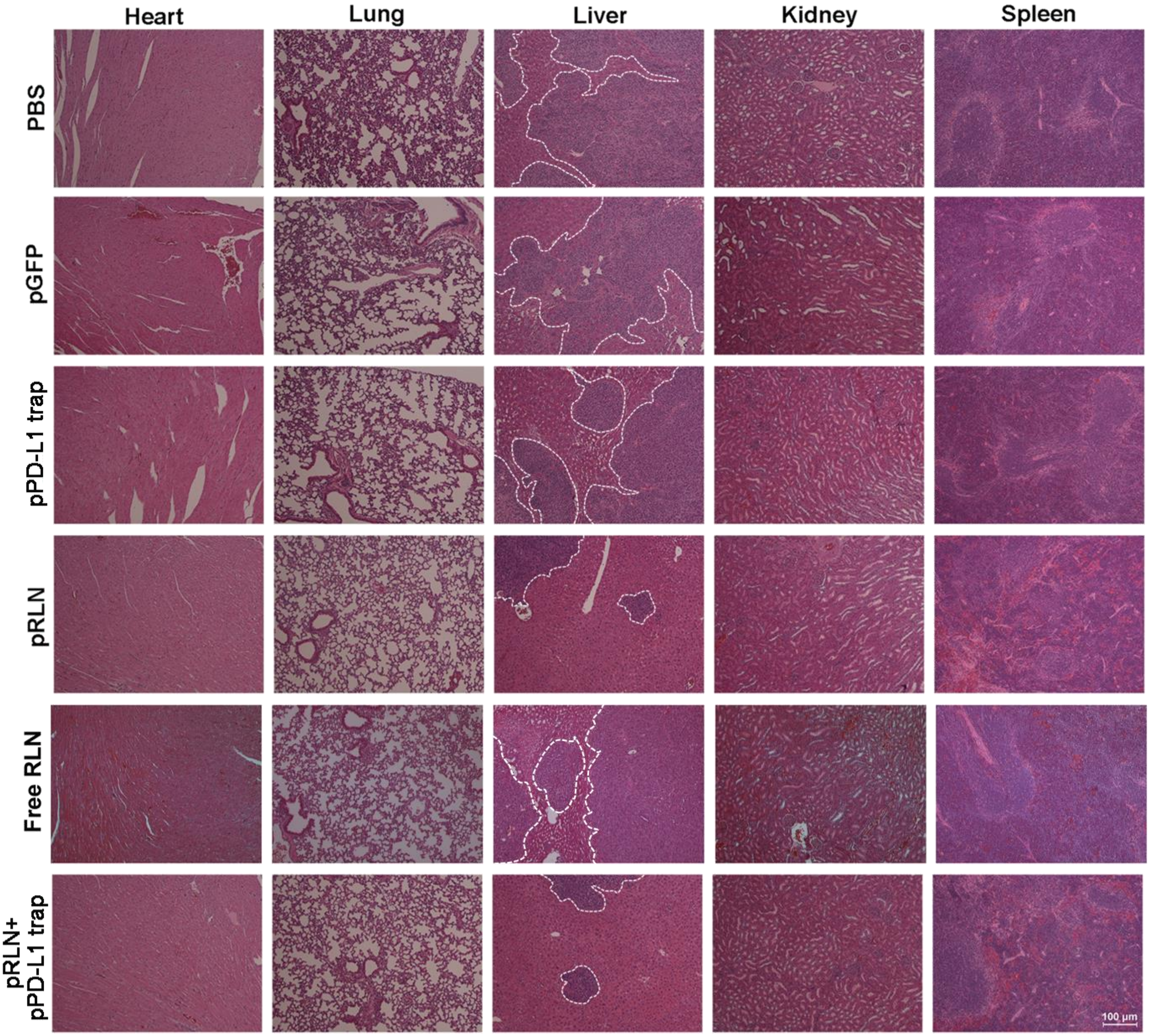

Supplementary Fig. 14 Pathological analyses of liver, spleen, heart, lung and kidney of CT26-FL3 liver metastasis bearing mice in the PBS, pGFP LCP, pPD-L1 trap LCP, free RLN, pRLN LCP and pRLN+pPD-L1 trap LCP treated groups on day 18. The regions in the white dotted lines are the liver metastatic lesions. Bar represents 100 µm.

Supplementary Figure 15

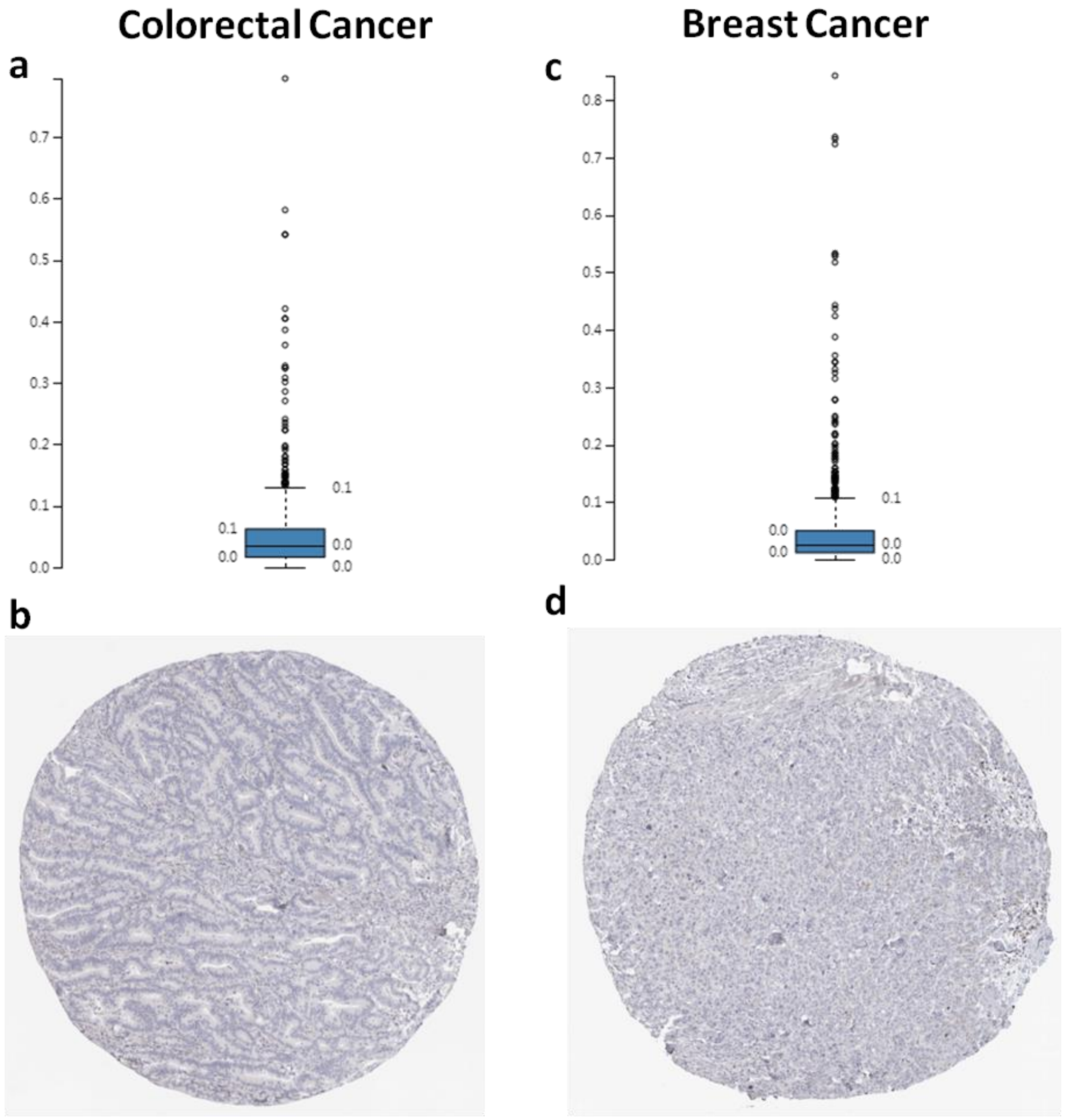

Supplementary Fig. 15 RXFP1 expression in human CRC and breast cancers. **(a), (c).** mRNA level of RXFP1 in CRC or breast cancer patient samples cited from TCGA database[1, 2]. **(b), (d).** Representative tumor tissue of CRC cancer patient sample (CRC-2948) or breast cancer patient sample (breast cancer-2174) cited from Swedish-based program-The Human Protein Atlas[1, 2].

Supplementary Table 1. Antibodies Used in the Study

Antibodies	Company	Catalog #	Application	Dilution
Alexa Fluor® 488 Anti-α-SMA	Invitrogen	53-9760-82	IF	1:500
Alexa Fluor®647 Anti-CD3	BioLegend	100209	IF	1:200
Anti-Sigma-1 receptor	Santa Cruz Biotechnology	sc-166392	IF	1:200
Anti-RXFP1	R & D Systems	MAB8898	IF	2 μg/mL
Alexa Fluor® 647 Goat Anti-Mouse IgG H&L	Abcam	ab150115	IF	1:500
Anti-α-SMA	Abcam	ab5694	WB	1:200
Anti-Collagen I	Abcam	Ab34710	WB	1:1000
Anti-pSMAD2	Cell Signaling Technology	18338	WB	1:1000
Anti-pSMAD3	Cell Signaling Technology	9520	WB	1:1000
Anti-NOS2	Santa Cruz Biotechnology	SC-7271	WB	1:400
Anti-SDF1 (CXCL12)	Abcam	Ab25117	WB	1:1000
GAPDH	Santa Cruz Biotechnology	SC-25778	WB	1:2000
Goat anti-rabbit HRP	Abcam	Ab205718	WB	1:2000
m-IgGκ BP-HRP	Santa Cruz Biotechnology	516102	WB	1:1000
PE Anti-CD8a	BD Pharmingen	553032	Flow	1:250
PerCP/Cy5.5 Anti-CD-4	BioLegend	100433	Flow	1:250
Alexa Fluor® 700 Anti-CD-3	BD Pharmingen	561388	Flow	1:250
PE-Cy7 Anti-CD25	ebioscience	25-0251-81	Flow	1:250
Alexa Fluor® 647 Anti-Foxp3	BD Pharmingen	560401	Flow	1:250
Alexa Fluor® 647 Anti-Ly-6G/Ly-6C (Gr-1)	BioLegend	108418	Flow	1:500
PerCP/Cy5.5 Anti-I-A^b	BioLegend	116415	Flow	1:250
FITC Anti-CD11C	ebioscience	11-0114-82	Flow	1:250
Alexa Fluor® 700 Anti-F4/80	BioLegend	123129	Flow	1:500
PE Anti-CD206	BioLegend	141705	Flow	1:250
APC anti-IL-17A	BioLegend	506915	Flow	1:250

Supplementary Table 2. Primers used in the study

Primer	Sequence or Catalog #	Assay method
Mouse GAPDH Forward	CGT CCC GTA GAC AAA ATG GT	SYBR ® Green
Mouse GAPDH Reverse	TCA ATG AAG GGG TCG TTG AT	SYBR ® Green
Mouse RXFP1 Forward	ACG AGC TGT CCC ATC AGT TT	SYBR ® Green
Mouse RXFP1 Reverse	ATG TGC TGA CAG AGG GGT TT	SYBR ® Green
Mouse GAPDH	Applied Biosystems (Mm99999915_g1)	TaqMan ®
Mouse Relaxin 1	Applied Biosystems (Mm01208503_m1)	TaqMan ®
Mouse CCL2	Applied Biosystems (Mm00441242_m1)	TaqMan ®
Mouse CCL5	Applied Biosystems (Mm01302427_m1)	TaqMan ®
Mouse IL4	Applied Biosystems (Mm00445259_m1)	TaqMan ®
Mouse IL6	Applied Biosystems (Mm00446190_m1)	TaqMan ®
Mouse IL10	Applied Biosystems (Mm01288386_m1)	TaqMan ®
Mouse IL12a	Applied Biosystems (Mm00434169_m1)	TaqMan ®
Mouse IFN-γ	Applied Biosystems (Mm01168134_m1)	TaqMan ®
Mouse TGF-β	Applied Biosystems (Mm01178820_m1)	TaqMan ®
Mouse PDGFb	Applied Biosystems (Mm00440677_m1)	TaqMan ®
Mouse PDGFc	Applied Biosystems (Mm00480205_m1)	TaqMan ®
Mouse FGF2	Applied Biosystems (Mm01285715_m1)	TaqMan ®

References:

1. Uhlen, M. et al. A pathology atlas of the human cancer transcriptome. *Science* **357**, eaan2507 (2017).

2. The Human Protein Atlas, https://www.proteinatlas.org/.
