# Supplementary material for: Relaxin gene delivery mitigates liver metastasis and synergizes with check point therapy
Source: Nat Commun. 2019 Jul 5;10:2993. doi: 10.1038/s41467-019-10893-8 (PMC6611764; doi:10.1038/s41467-019-10893-8)
Supplement: Supplementary file 3 — Reporting Summary [file 41467_2019_10893_MOESM3_ESM.pdf]

nature research

Corresponding author(s): Leaf Huang

Last updated by author(s): May 10, 2019

Reporting Summary

Nature Research wishes to improve the reproducibility of the work that we publish. This form provides structure for consistency and transparency in reporting. For further information on Nature Research policies, see Authors & Referees and the Editorial Policy Checklist.

Statistics

For all statistical analyses, confirm that the following items are present in the figure legend, table legend, main text, or Methods section.

n/a	Confirmed	
☐	☒	The exact sample size (*n*) for each experimental group/condition, given as a discrete number and unit of measurement
☐	☒	A statement on whether measurements were taken from distinct samples or whether the same sample was measured repeatedly
☐	☒	The statistical test(s) used AND whether they are one- or two-sided<br>*Only common tests should be described solely by name; describe more complex techniques in the Methods section.*
☐	☒	A description of all covariates tested
☐	☒	A description of any assumptions or corrections, such as tests of normality and adjustment for multiple comparisons
☐	☒	A full description of the statistical parameters including central tendency (e.g. means) or other basic estimates (e.g. regression coefficient) AND variation (e.g. standard deviation) or associated estimates of uncertainty (e.g. confidence intervals)
☐	☒	For null hypothesis testing, the test statistic (e.g. *F*, *t*, *r*) with confidence intervals, effect sizes, degrees of freedom and *P* value noted<br>*Give P values as exact values whenever suitable.*
☒	☐	For Bayesian analysis, information on the choice of priors and Markov chain Monte Carlo settings
☒	☐	For hierarchical and complex designs, identification of the appropriate level for tests and full reporting of outcomes
☐	☒	Estimates of effect sizes (e.g. Cohen's *d*, Pearson's *r*), indicating how they were calculated

Our web collection on statistics for biologists contains articles on many of the points above.

Software and code

Policy information about availability of computer code

Data collection	Applied Biosystems® 7500 fast and 7500 Real-Time PCR System (7500 v2.3) was used to collect relative gene expression (RQ) values. Living Imaging 4.5.5* was used to collect and quantify bioluminescence signals for in vivo monitoring of liver metastasis progression, ex vivo liver metastasis burden, and fluorescence signals for biodistribution study. Zeiss ZEN 2011 was used to collect confocal images. Image Lab 6.0.1 was used to acquire western blot images. Flow cytometry data were acquired with BD FACSDIVA™.
Data analysis	Flow cytometry data were analyzed with FlowJo V10. ImageJ 1.48v was used for confocal and western blot image processing. GraphPad Prism 7.04v was used to analyze the data.

For manuscripts utilizing custom algorithms or software that are central to the research but not yet described in published literature, software must be made available to editors/reviewers. We strongly encourage code deposition in a community repository (e.g. GitHub). See the Nature Research guidelines for submitting code & software for further information.

Data

Policy information about availability of data

All manuscripts must include a data availability statement. This statement should provide the following information, where applicable:

- Accession codes, unique identifiers, or web links for publicly available datasets
- A list of figures that have associated raw data
- A description of any restrictions on data availability

The authors declare that the data supporting the findings of this study are available within the paper and its supplementary information files.

Field-specific reporting

Please select the one below that is the best fit for your research. If you are not sure, read the appropriate sections before making your selection.

☒ Life sciences ☐ Behavioural & social sciences ☐ Ecological, evolutionary & environmental sciences

For a reference copy of the document with all sections, see nature.com/documents/nr-reporting-summary-flat.pdf

Life sciences study design

All studies must disclose on these points even when the disclosure is negative.

Sample size	No sample-size calculations were performed. Sample size was determined to be adequate based on the magnitude and consistency of measurable differences between groups.
Data exclusions	No exclusion criteria were incorporated in the design of the experiments for this study.
Replication	For each series of experiments, all replication attempts were successful.
Randomization	Online Methods, 'Metastatic growth inhibition assay' section: "Mice bearing liver metastases were randomized blindly into different treatment groups".
Blinding	Online Methods, 'Metastatic growth inhibition assay' section: "the investigator was blinded to the group allocation during the animal experiments." And all analyses were performed by an investigator blinded to the experimental conditions.

Reporting for specific materials, systems and methods

We require information from authors about some types of materials, experimental systems and methods used in many studies. Here, indicate whether each material, system or method listed is relevant to your study. If you are not sure if a list item applies to your research, read the appropriate section before selecting a response.

Materials & experimental systems

n/a	Involved in the study	
☐	☒	Antibodies
☐	☒	Eukaryotic cell lines
☒	☐	Palaeontology
☐	☒	Animals and other organisms
☒	☐	Human research participants
☒	☐	Clinical data

Methods

n/a	Involved in the study	
☒	☐	ChIP-seq
☐	☒	Flow cytometry
☒	☐	MRI-based neuroimaging

Antibodies

Antibodies used	The antibodies used in this study were summarized in Supplementary Table 1. The primers for quantitative PCR used in this study were summarized in Supplementary Table 2.
Validation	All details were provided within the Online Methods and Supplementary Table 1 and Supplementary Table 2, including species, catalog numbers, manufactures, and citations when possible.

Eukaryotic cell lines

Policy information about cell lines

Cell line source(s)	All details were provided within the Online Methods, 'cell lines' section.
Authentication	None of the cell lines used were authenticated
Mycoplasma contamination	The cell lines were not tested for mycoplasma contamination
Commonly misidentified lines (See ICLAC register)	No cell lines used are listed in the database of commonly misidentified cell lines.

Animals and other organisms

Policy information about studies involving animals; ARRIVE guidelines recommended for reporting animal research

Laboratory animals	Description of research mice used for experiments can be found in the relevant figure legends and Online Methods, 'Mouse model establishment' section.
Wild animals	The study did not involve wild animals.
Field-collected samples	The study did not involve samples collected from the filed.
Ethics oversight	All animal handling procedures were approved by the University of North Carolina at Chapel Hill's Institutional Animal Care and Use Committee.

Note that full information on the approval of the study protocol must also be provided in the manuscript.

Flow Cytometry

Plots

Confirm that:

☒ The axis labels state the marker and fluorochrome used (e.g. CD4-FITC).

☒ The axis scales are clearly visible. Include numbers along axes only for bottom left plot of group (a 'group' is an analysis of identical markers).

☒ All plots are contour plots with outliers or pseudocolor plots.

☒ A numerical value for number of cells or percentage (with statistics) is provided.

Methodology

Sample preparation	Sample preparation listed in Online Methods, 'Flow cytometry assay' section.
Instrument	BD LSR II, LSRFortessa
Software	BD FACSDIVA™
Cell population abundance	Flow cytometry was used for quantification purposes only (i.e. no postsorting fractions were collected)
Gating strategy	For all experiments FSC-A/ SSC-A gates of the starting cell population were used to discriminate between viable cells and cell debris. Singlet and doublet cells were discriminated using FSC-A/ FSC-W gating. Isotype control stained cells were used to distinguish between background staining and specific antibody staining.

☒ Tick this box to confirm that a figure exemplifying the gating strategy is provided in the Supplementary Information.
